# Patient-reported outcomes 3 and 18 months after mastectomy and immediate prepectoral implant-based breast reconstruction in the UK Pre-BRA prospective multicentre cohort study

**DOI:** 10.1093/bjs/znaf032

**Published:** 2025-02-25

**Authors:** Kate L Harvey, Leigh Johnson, Parisa Sinai, Nicola Mills, Paul White, Christopher Holcombe, Shelley Potter, The Pre-BRA Feasibility Study Steering Group, Peter Barry, Simon Cawthorn, Matthew Gardiner, Gareth Irwin, Cliona Kirwan, Mairead McKenzie, Shireen McKenzie, Rachel O’Connell, Georgette Oni, Tim Rattay, Pankaj Roy, Joanna Skillman, Soni Soumian, Raghavan Vidya, Lisa Whisker, Samantha Williams

**Affiliations:** National Institute for Health Research Bristol Biomedical Research Centre, University Hospitals Bristol and Weston NHS Foundation Trust, and University of Bristol, Bristol, UK; National Institute for Health Research Bristol Biomedical Research Centre, University Hospitals Bristol and Weston NHS Foundation Trust, and University of Bristol, Bristol, UK; National Institute for Health Research Bristol Biomedical Research Centre, University Hospitals Bristol and Weston NHS Foundation Trust, and University of Bristol, Bristol, UK; Population Health Sciences, Bristol Medical School, Bristol, UK; Applied Statistics Group, University of the West of England, Bristol, UK; Breast Unit, Royal Liverpool University Hospital, Liverpool, UK; National Institute for Health Research Bristol Biomedical Research Centre, University Hospitals Bristol and Weston NHS Foundation Trust, and University of Bristol, Bristol, UK; Bristol Breast Care Centre, North Bristol NHS Trust, Bristol, UK

## Abstract

**Introduction:**

Prepectoral techniques are becoming standard of care for implant-based breast reconstruction due to reduced impact on chest wall function and improved patient satisfaction. Evidence to support these benefits, however, is lacking. Here, patient-reported outcomes (PROs) of prepectoral breast reconstruction (PPBR) in the Pre-BRA cohort are reported.

**Methods:**

Women undergoing PPBR after mastectomy for breast cancer or risk reduction between July 2019 and December 2020 were recruited. Participants completed the BREAST-Q preoperatively and at 3 and 18 months following surgery together with a single item evaluating overall satisfaction at 18 months. Women completing at least one BREAST-Q scale at any timepoint were eligible for inclusion. Questionnaires were scored according to the developers’ instructions and scores compared over time. Exploratory analysis, adjusting for baseline scores was performed to explore factors impacting PROs.

**Results:**

In total 338 of 343 (98.5%) women undergoing PPBR at 40 UK centres were included in the analysis. Compared with baseline scores, women reported statistically significant and clinically meaningful decreases in both ‘Physical’ and ‘Sexual well-being’ at 3 and 18 months. Adjusting for baseline, at 18 months, those experiencing implant loss or having surgery for malignancy reported lower scores in all BREAST-Q domains. Overall, two-thirds of women (167/251) rated the outcome of their reconstruction as ‘excellent/very good’, but experiencing major complications, implant loss, and being dissatisfied with wrinkling/rippling in the reconstructed breast were associated with reduced satisfaction.

**Conclusions:**

PPBR impacts postoperative physical well-being and PROs are variable. These findings should be discussed with patients to support informed decision-making based on realistic expectations of outcome.

**Study registration:**

ISRCTN11898000.

## Teaser

Prepectoral implant-based breast reconstruction is rapidly becoming adopted into practice due to perceived improvements in patient-reported outcomes (PROs). This multicentre study of 338 women having prepectoral reconstruction showed that compared with preoperative scores, physical well-being scores at 3 and 18 months following surgery were significantly lower. This study suggests the proposed benefits of prepectoral reconstruction are not realized. These results should be shared with women considering surgery to ensure they have realistic expectations of outcome.

## Introduction

Up to 40%^1^ of the 55 000^2^ women diagnosed with breast cancer each year in the UK will undergo a mastectomy as part of their treatment. Mastectomy can profoundly impact women’s well-being and the National Institute of Health and Care Excellence recommends that breast reconstruction should be routinely offered to improve quality of life^3^.

Implant-based reconstruction is the most performed reconstructive procedure worldwide^4,5^ and over the last decade, the technique has evolved rapidly despite a lack of high-quality evidence to support best practice. Traditionally, a two-stage procedure was performed, with initial placement of a tissue expander under the pectoralis muscle. After gradual expansions a second operation was performed to insert a definitive fixed-volume implant. The introduction of biological and synthetic meshes allowed the creation of a larger subpectoral pocket at the initial operation, thus allowing a fixed-volume implant to be inserted in a single stage. The use of mesh allowed women to avoid a second operation and was perceived to result in better cosmetic outcomes through improved lower pole projection. Subpectoral mesh-assisted reconstruction therefore rapidly became standard of care despite a lack of high-quality evidence to support the proposed benefits^6–11^.

Most recently, the technique has evolved so that the implant, usually supported by mesh, is placed on top rather than underneath the pectoralis muscle^12^. This prepectoral technique avoids ‘implant animation’, the upwards movement of the implant seen when the chest wall muscles contract. It is also hypothesized to have less impact on chest wall function as the pectoralis muscle is not lifted, and to lead to better cosmetic outcomes than subpectoral techniques as the implant is placed in a more anatomical position^12^. Subcutaneous implant-based reconstruction, however, was previously abandoned by the reconstructive community due to unacceptable complication rates^13–16^. High-quality evidence is therefore needed not only to demonstrate that the technique is safe, but also that the proposed improvements in patient-centred outcomes are realized.

Although there is a rapidly growing body of evidence to suggest that the complication rates of pre- and subpectoral reconstruction are broadly comparable^17,18^, studies exploring the hypothesized improvements in patient-reported outcomes (PROs) following prepectoral breast reconstruction (PPBR) remain scarce. Current evidence is limited to often small^19–23^, single-centre^24^ studies that only assess PROs at a single timepoint, thus limiting their value. Few studies have assessed how PROs change over time and these results are conflicting^25,26^. As breast reconstruction is performed to improve quality of life, high-quality multicentre studies evaluating the longitudinal impact of PPBR on PROs are urgently needed.

Pre-BRA is a multicentre prospective study^27^ that aimed to establish the safety and effectiveness of PPBR to inform the design of a future trial comparing pre- and subpectoral techniques. Here, the impact of PPBR on patient-reported outcomes at 3 and 18 months following surgery in women participating in the Pre-BRA study is reported.

## Methods

### Study design and participants

Pre-BRA was a single-arm, multicentre, IDEAL Phase 2a/2b prospective observational cohort study with embedded qualitative methods^27^. Full ethical approval was obtained (NRES OXFORD-B South Central Committee Ref:19/SC/0129. IRAS ID: 255421) and the study was prospectively registered prior to commencing participant recruitment (ISRCTN11898000). The short-term safety outcomes have been reported elsewhere^28^. The study methods have been described in detail elsewhere^27,28^, but in brief: women aged 16 or over undergoing mastectomy for malignancy or risk-reduction who elected to undergo immediate implant-based reconstruction and who were considered technically suitable for a prepectoral procedure by their operating surgeon were eligible to participate in the study.

### Procedures

Eligible patients were prospectively identified from multidisciplinary meetings, clinics and operating lists at participating centres and given a study information sheet by their local surgical teams. Patients who elected to participate provided written consent and completed baseline PRO questionnaires. Baseline demographic data were collected *via* electronic case report forms (CRFs) hosted on REDCap^29^.

All participants underwent immediate prepectoral implant-based reconstruction as per local practice. Type of mastectomy (skin-sparing, nipple-sparing, or skin-reducing), choice of implant (tissue expander, fixed-volume implant or adjustable implant), and use and type of mesh (biological or synthetic) were as per surgeon and patient preference. Raising the pectoralis muscle was prohibited but if, intraoperatively, the operating surgeon felt that prepectoral reconstruction was not possible for safety reasons (for example poor-quality skin flaps) and the preoperative surgical plan was modified, the modification (for example conversion to subpectoral reconstruction) and rationale were captured on the operative CRF. Use of antibiotics, drains, and dressings were as per local practice.

All complications were defined a priori using standardized definitions^10,30,31^ and complication and oncological data were collected by clinical, or case note review at 30 days and 3 months as per local practice.

### Outcome measures

Participants were asked to complete the validated BREAST-Q questionnaire^32^ either electronically or on paper as per patient preference at 3 and 18 months post surgery. At 18 months, participants were also asked to rate the overall outcome of their reconstruction on a 5-point Likert scale (excellent/very good/good/fair/poor) consistent with the evaluation in the UK National Mastectomy and Breast Reconstruction Audit^33,34^ and other large-scale UK-based studies^10^ and to report the receipt of any additional unplanned surgical procedures, in particular unplanned implant removal.

#### BREAST-Q questionnaire

The BREAST-Q is a validated questionnaire robustly developed for patients undergoing breast reconstruction^32,35^. It consists of four main scales: ‘Satisfaction with breasts’, ‘Physical well-being: chest’, ‘Sexual well-being’, and ‘Psychosocial well-being’. Each scale is Rasch-transformed to give a score out of 100, with higher scores reflecting better outcomes. A four-point difference in ‘Satisfaction with breast’, ‘Psychosocial’ and ‘Sexual well-being’ domains and a three-point difference in the ‘Physical well-being: chest’ domain represent the minimum clinically important difference^36^.

Two additional BREAST-Q scales with particular relevance to PPBR were also evaluated at 3 and 18 months post surgery: the two-item ‘Satisfaction with implants’ scale evaluating satisfaction with wrinkles/ripples that were visible and/or palpable in the reconstructed breast and the newly developed 12-item ‘Animation deformity’ scale, designed to assess the impact of chest wall animation on quality of life^37^.

### Sample size and statistical analysis

#### Sample size considerations

The Pre-BRA study was powered to detect an unacceptable implant loss rate of >9% at 3 months^27,28^. This was based on the implant loss rate observed in the UK iBRA study that included over 2000 implant-based reconstructions from 81 centres^10^. The PRO study was exploratory and hypothesis generating so no formal sample size calculation was performed.

#### Statistical analysis

Pre-BRA participants were included in the PRO analysis if they had completed at least one of the main BREAST-Q scales at any timepoint. Those who did not meet this criterion were excluded.

Simple summary statistics were calculated to describe demographic, procedure, and outcome data. Categorical data were summarized by counts and percentages and continuous data by mean and standard deviation.

The BREAST-Q questionnaire was scored according to the developers’ instructions and unadjusted scores at 3 and 18 months compared with baseline values using a paired *t*-test. Linear regression, adjusting for baseline scores was used to explore patient, procedure, and treatment-related factors hypothesized to impact PROs at 18 months based on the literature^38–41^ and expert opinion. These included age (<45 years *versus* older), BMI (>30 *versus* <30), smoking status (current *versus* non-smoker), co-morbidities (yes/no); indication for surgery (malignancy *versus* risk reduction), unilateral *versus* bilateral reconstruction, nipple preservation *versus* not, high mastectomy weights (>600 g *versus* <600 g), receipt of chemotherapy or radiotherapy and experiencing any complication, major complications requiring readmission or re-operation at 3 months or implant loss at any timepoint.

For implant reconstruction specific domains only measured post surgery, 3 and 18 month scores were calculated and compared. The ‘Satisfaction with implants’ scale does not have formal scoring guidance so the scores for each item was dichotomized into ‘Very/Somewhat satisfied’ *versus* ‘Very/Somewhat dissatisfied’ and the proportions of women reporting dissatisfaction with the look and/or feel of ripples/wrinkles in their reconstructed breast at each timepoint compared using chi-squared statistics.

The single item on overall satisfaction with the outcome of surgery was dichotomized into ‘excellent/very good’ *versus* ‘good/fair/poor’ consistent with the approach used in previous studies to facilitate comparison of techniques^42,43^ and an exploratory univariable analysis using chi-squared statistics performed to explore whether the hypothesized factors listed above impacted overall satisfaction.

## Results

Of the 347 women planned for PPBR at 40 UK sites between 1 July 2019 and 31 December 2020, 343 successfully underwent the procedure. Prepectoral reconstruction was abandoned intraoperatively in four patients due to concerns about skin-flap viability. Of the 343 women undergoing PPBR, 326/343 (95.0%), 296/343 (86.3%), and 255/343 (74.3%) women completed at least one main BREAST-Q scale at baseline, 3 and 18 months respectively, and a total of 338/343 (98.5%) were eligible for inclusion in the analysis.

Demographics of the PRO cohort are summarized in *Table 1*. The mean age of participants was 48.6 years (s.d. 10.9). Most women had surgery for malignancy (*n* = 295, 87.3%) and a quarter (*n* = 84, 24.9%) had bilateral reconstruction. Approximately 40% of participants experienced at least one postoperative complication at 3 months (*n* = 141, 41.7%) and 20% (*n* = 68) had a major complication requiring readmission and/or re-operation. In addition to the 27 women who had experienced implant loss by 3 months, a further 12 self-reported having their implant removed between 3 and 18 months. A total of 39 women (11.6%) therefore experienced implant loss in the cohort overall. Women who experienced a major complication or lost their implant were significantly less likely to complete the 18 month questionnaire (38/68 (55.9%) major complications *versus* 217/270 (80.4%) no complications, *P* < 0.001; 20/39 (51.3%) implant loss *versus* 235/299 (78.6%) no implant loss, *P* < 0.001). Of the 20 patients reporting an implant loss who completed the 18-month questionnaire, 9 (45.0%) reported having a secondary reconstruction with either an implant (*n* = 7) or an autologous (*n* = 2) procedure.

**Table 1:** Demographics of the Pre-BRA PROMs cohort

	*N* = 338
**Age, years (mean,sd)**	48.6 (10.9)
**Age group**	
<45	125 (37.0)
45–59	158 (46.8)
60 and over	55 (16.3)
**BMI**	
Underweight <18.5	3 (0.9)
Normal 18.5–24.9	167 (49.4)
Overweight 25–29.9	91 (26.9)
Obese >30	68 (20.1)
Not reported	9 (2.7)
**Smoking**	
Smoker	40 (11.8)
Non-smoker	295 (87.3)
Not reported	3 (0.9)
**Laterality**	
Unilateral	254 (75.2)
Bilateral	84 (24.9)
**Indication for surgery**	
Malignancy	295 (87.3)
Risk reduction	43 (12.7)
**Co-morbidity**	
Yes	84 (24.9)
No	244 (72.2)
Not reported	10 (3.0)
**Nipple-sparing procedure**	
At least one	179 (53.0)
Nipple sacrificing	158 (46.8)
Not reported	1 (0.3)
**Complications at 3 months**	
Any complication	141 (41.7)
Implant loss	27 (8.0)
Major complications requiring readmission or re-operation	68 (20.1)
Infection	65 (19.2)
Implant loss at any time point*	39 (11.6)
**Chemotherapy**	
Yes	67 (19.9)
Not required	209 (61.8)
Already received	44 (13.0)
Not reported	18 (5.3)
**Radiotherapy**	80 (23.7)
**Endocrine therapy**	181 (53.6)
**PROMs completion†**	
Baseline	326 (96.4)
3 months	296 (87.6)
18 months	255 (75.4)

^*^Includes 12 women self-reporting implant loss at 18 months. †At least one BREAST-Q scale.

### Main BREAST-Q scales

**Fig. 1:**
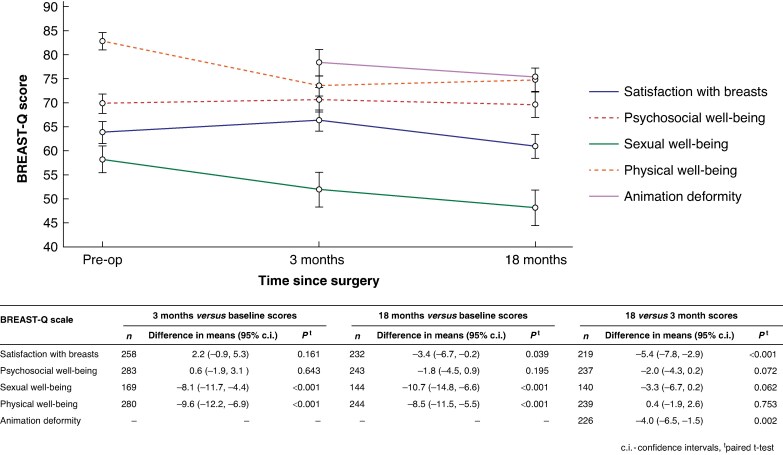
Changes in unadjusted BREAST-Q scores over time

Unadjusted BREAST-Q scores at baseline, 3, and 18 months following surgery are summarized in *Fig. 1* and *Table S1*. Compared with scores at baseline, women reported statistically significant and clinically meaningful decreases in both their ‘Physical well-being: chest’ (baseline to 3 months −9.6 (95% c.i. −12.2, −6.9), *P* < 0.001; baseline to 18 months −8.5 (95% c.i. −11.5, −5.5), *P* < 0.001) and ‘Sexual well-being’ (baseline to 3 months −8.1 (95% c.i. −11.7, −4.4), *P* < 0.001; baseline to 18 months −10.7 (95% c.i. −14.8, −6.6), *P* < 0.001) scores at 3 and 18 months after surgery, but no significant changes were seen between 3 and 18 months (*Fig. 1*). There were no significant changes in ‘Satisfaction with breasts’ scores between baseline and 3 months and only borderline decreases in scores between baseline and 18 months. Statistically significant and clinically meaningful decreases in ‘Satisfaction with breasts’ scores were, however, seen between 3 and 18 months in women completing the questionnaire at both timepoints (−5.4, 95% c.i. −7.8, −2.9, *P* < 0.001). No other significant changes were seen (*Fig. 1*).

Exploratory analyses of factors impacting 18-month BREAST-Q scores, adjusting for baseline are summarized in *Table 2*. Women having PPBR for risk reduction reported significantly higher scores across all domains than those having surgery for malignancy. Women with larger mastectomy specimens reported relatively better ‘Psychosocial well-being’ compared with those whose mastectomy weights were lower, and those having bilateral reconstruction reported better ‘Sexual well-being’ than those having unilateral surgery. By contrast, women receiving radiotherapy and those with BMIs >30 reported worse ‘Physical well-being: chest’ scores at 18 months. Experiencing implant loss had the most marked impact on PROs, with decreases of 19–23 points across all BREAST-Q domains at 18 months (*Table 2*).

**Table 2:** Exploratory analysis of factors impacting 18-month BREAST-Q scores, adjusting for baseline

	Psychosocial well-being, *n* = 251		Physical well-being chest, *n* = 252		Sexual well-being, *n* = 164		Satisfaction with breasts *n* = 245	
	Coefficient (95% c.i.)	*P*	Coefficient (95% c.i.)	*P*	Coefficient (95% c.i.)	*P*	Coefficient (95% c.i.)	*P*
**Age**								
<45	Reference		Reference		Reference		Reference	
45–59	−1.06 (−6.65, 4.53)	0.710	−3.62 (−9.14, 1.90)	0.198	−7.63 (−15.36, 0.10)	0.053	−0.32 (−6.98, 4.32)	0.644
60 and over	4.07 (−4.47, 10.62)	0.423	−2.51 (−9.82, 4.79)	0.499	−7.68 (−20.62, 5.26)	0.243	−0.59 (−8.14, 6.96)	0.878
**BMI**								
>30	Reference		Reference		Reference		Reference	
≤30	−5.99 (−12.69, 0.71)	0.079	−11.40 (−17.82,−4.99)	0.001	−5.26 (−15.87, 5.34)	0.328	−5.14 (−12.07, 1.78)	0.145
**Smoking**								
No	Reference		Reference		Reference		Reference	
Yes	−8.50 (−17.02, 0.02)	0.051	−7.46 (−15.81,0.89)	0.080	−0.26 (−12.46, 11.95)	0.967	−9.66 (−18.32,−0.99)	0.029
**Laterality**								
Bilateral	Reference		Reference		Reference		Reference	
Unilateral	1.90 (−3.65, 7.46)	0.501	−0.64 (−4.94, 6.21)	0.823	12.77 (5.20, 20.35)	0.001	1.98 (−3.61, 7.47)	0.486
**Indication for surgery**								
Risk reduction	Reference		Reference		Reference		Reference	
Malignancy	10.94 (3.64, 18.23)	0.003	10.09 (2.90, 17.27)	0.006	15.51 (5.66, 25.37)	0.002	8.65 (1.41, 15.90)	0.019
**Co-morbidity**								
No	Reference		Reference		Reference		Reference	
Yes	−3.34 (−9.22, 2.55)	0.266	1.12 (−4.73, 6.97)		−0.31 (−9.13, 8.50)	0.944	1.95 (−4.13, 8.05)	0.527
**Nipple-sparing procedure**								
No	Reference		Reference		Reference		Reference	
Yes	3.37 (−1.68, 8.42)	0.190	−3.58 (−8.58, 1.43)	0.160	0.26 (−7.19, 7.72)	0.944	−4.59 (−9.68, 0.51)	0.077
**Mastectomy Weight**								
<600 g	Reference		Reference		Reference		Reference	
>600 g	8.81 (2.57, 15.06)	0.006	6.55 (0.19, 12.92)	0.044	10.24 (0.34, 20.15)	0.043	−5.72 (−0.90, 12.33)	0.090
**Chemotherapy**								
None	Reference		Reference		Reference		Reference	
Neoadjuvant	−1.22 (−9.17, 6.73)	0.763	−5.35 (−13.05, 2.36)	0.173	−4.96 (−15.37, 5.46)	0.348	−6.29 (−14.47, 1.88)	0.131
Adjuvant	−3.07 (−9.40, 3.26)	0.341	−7.90 (−14.22, −1.57)	0.015	−5.78 (−15.06, 3.501)	0.221	−7.31 (−13.72, −0.90)	0.026
**Radiotherapy**								
No	Reference		Reference				Reference	
Yes	−5.16 (−11.08,0.76)	0.087	−9.33 (−15.04, −3.61)	0.001	−4.66 (−13.53, 4.21)	0.301	−3.70 (−9.78, 2.38)	0.232
**Any complication**								
No	Reference		Reference		Reference		Reference	
Yes	−2.36 (−7.61,2.88)	0.376	−2.90 (−8.04, 2.24)	0.269	3.77 (−4.06, 11.60)	0.352	−1.87 (−7.12, 3.39)	0.485
Major complication	−3.03 (−10.37, 4.30)	0.416	−4.08 (−11.34, 3.18)	0.269	−0.38 (−12.05, 11.28)	0.948	−7.26 (−14.51, 0.00)	0.050
**Implant loss at any timepoint**								
No	Reference		Reference				Reference	
Yes	−20.98 (−30.61,−11.35)	<0.001	−20.54 (−29.81, −11.26)	<0.001	−18.97 (−32.39, −5.54)	0.006	−22.83 (−32.51. −13.16)	<0.001

### Implant-specific BREAST-Q scales

At 3 months, approximately a third of women (94/273, 34.8%) reported dissatisfaction with the ripples/wrinkles in their reconstructed breast, but this proportion increased significantly to 45.6% (111/244) by 18 months (*P* < 0.001). Unadjusted scores for ‘Animation deformity’ decreased from 78.4 (95% c.i. 76.0, 80.9) at 3 months to 75.4 (95% c.i. 72.6, 78.2) at 18 months. When scores were compared in women completing the scale at both timepoints, a statistically significant decrease of 4.0 points (95% c.i. −6.5 to −1.5, *P* = 0.002) was seen (*Fig. 1*).

### Overall satisfaction with the outcome of reconstruction

At 18 months, two-thirds of women (167/251, 66.5%) rated the outcome of their reconstruction as ‘excellent’ or ‘very good’. In the exploratory analysis, only experiencing a major complication, implant loss, and dissatisfaction with ripples/wrinkles in the reconstructed breast were associated with reduced overall satisfaction at 18 months. Women with mastectomy weights >600 g were more satisfied with the outcome of their surgery than those whose mastectomy weights were smaller (*Table 3*).

**Table 3:** Exploratory analysis of factors impacting overall satisfaction with reconstruction at 18 months

	Excellent/very good	Good/fair/poor	*P* *
	(*n* = 167, 66.5%)	(*n* = 84, 33.5%)	
**Age**			
<45	66 (39.5)	25 (29.8)	0.253
45–59	75 (44.9)	41 (48.8)	
60 and over	26 (15.6)	18 (21.4)	
**BMI**			
>30	139 (83.2)	61 (72.6)	0.057
≤30	24 (14.4)	20 (23.8)	
Not reported	4 (2.4)	3 (3.6)	
**Smoking**			
Yes	13 (7.8)	11 (13.1)	0.172
No	153 (91.6)	72 (85.7)	
Not reported	1 (1.1)	1 (1.2)	
Bilateral reconstruction	46 (27.5)	27 (32.1)	0.499
**Indication for surgery**			
Malignancy	144 (86.2)	74 (88.1)	0.679
Risk reduction	23 (13.8)	10 (11.9)	
**Co-morbidities**			
Yes	37 (22.2)	27 (32.1)	0.085
No	124 (74.3)	54 (64.3)	
Not reported	6 (3.6)	3 (3.6)	
**Nipple-sparing procedure**			
Yes	88 (52.7)	47 (56.0)	0.66
No	78 (46.7)	37 (44.1)	
Not reported	1 (0.6)	0 (0)	
Mastectomy weight			
<600 g	24 (14.4)	23 (27.4)	0.008
>600 g	137 (82.0)	55 (65.5)	
Not reported	6 (3.6)	6 (7.1)	
**Chemotherapy**			
None	108 (64.7)	48 (57.1)	0.164
Neoadjuvant	19 (11.4)	13 (15.5)	
Adjuvant	29 (17.4)	23 (27.4)	
**Not reported**	11 (6.7)	0 (0)	
**Radiotherapy**	33 (19.8)	23 (27.4)	0.171
**Any complication**	58 (34.7)	35 (41.7)	0.283
**Major complication**	19 (11.4)	18 (21.4)	0.034
**Implant loss at any timepoint**	5 (3.0)	13 (15.5)	<0.001
**Dissatisfied with look or feel of ripples/wrinkles in the reconstructed breast at 18 months**			
Yes	51 (30.5)	60 (76.9)	<0.001
No	113 (67.7)	18 (21.4)	
Not reported	3 (1.8)	6 (7.1)	

^*^Chi-squared test.

## Discussion

This is the first prospective multicentre study to evaluate the longitudinal impact of PPBR on PROs using a validated questionnaire. It suggests that contrary to the perceived benefits of the technique over subpectoral reconstruction, women undergoing PPBR report statistically significant and clinically meaningful decreases in their ‘Physical well-being: chest’ scores following surgery even though their pectoralis muscle is not raised. Undergoing surgery for malignancy (rather than risk reduction) and experiencing implant loss resulted in significantly lower BREAST-Q scores in all domains in the exploratory analysis. Implant loss rates were also a third higher than previously reported^28^ as 12 women self-reported having their implant removed between 3 and 18 months. Indeed, the true proportion of women experiencing implant loss is likely to be even higher than the 11.6% reported here, as these delayed implant loss were identified by patient self-report and women who lose their implant have been shown to be significantly less likely to complete follow-up PRO questionnaires in similar studies^42^, raising further safety concerns regarding the technique^28^.

Unanticipated issues with PPBR included women reporting ongoing concerns with ‘implant animation’ despite prepectoral implant placement. Indeed, ‘Animation deformity’ scores decreased significantly between 3 and 18 months, suggesting these issues worsened over time. In addition, 45% of women reported dissatisfaction with the look or feel of ripples/wrinkles in their reconstructed breast at 18 months compared with a third at 3 months, suggesting that this issue became more problematic as the postoperative swelling and seroma resolved. Two-thirds of women, however, rated the outcome of their reconstruction as either ‘Very good’ or ‘Excellent’, but experiencing major complications, implant loss, and/or being dissatisfied with the wrinkles/ripples in the reconstructed breast were associated with significantly lower levels of satisfaction overall.

It is unclear why PPBR, which does not disturb the pectoralis muscle, results in such significant reductions in ‘Physical well-being: chest’ score at 3 and 18 months. One explanation may be that all women in the cohort underwent skin/nipple-sparing or skin-reducing mastectomy and immediate implant reconstruction so they could experience tightness and discomfort from surgical scarring, even though the pectoral muscle was not raised. Indeed, although the UK multicentre iBRA PRO study suggested PPBR may be associated with improved ‘Satisfaction with breasts’ compared with subpectoral techniques, the number of patients undergoing PPBR was small (*n* = 14) and unadjusted scores in this larger cohort of women undergoing exclusively PPBR are almost identical to those in the iBRA cohort, most of whom underwent subpectoral mesh-assisted procedures^42^. Patient-reported outcomes after pre- and subpectoral reconstruction have been shown to be equivalent in other longitudinal studies^26^, but high-quality PRO studies are limited and systematic reviews comparing PROs in patients undergoing pre- and subpectoral procedures have generated conflicting results^17,44^. There is therefore an urgent need for high-quality multicentre studies to explore the longer-term impact of PPBR on PROs. This is vital as current data suggest that PPBR does not translate into improved PROs compared with other types of implant-based procedures^42^. As these outcomes are consistently poorer than those seen after autologous reconstruction^41^ or oncoplastic breast-conserving surgery^45^, these findings should be shared with women considering PPBR to ensure they are fully informed about the likely outcomes of their surgery.

This study provided much-needed data regarding the impact of PPBR on PROs, but it has limitations that require consideration. This is a single-arm study, so it is not possible to directly compare the PROs of PPBR with other IBBR techniques. Prepectoral reconstruction, however, has now become the standard of care in the UK, so a comparative study would be challenging. Response bias is a key concern in PRO studies with ethnicity and sociodemographic status shown to impact response rates^39,46^. In addition, as previously reported^42^, women experiencing major complications and implant loss were significantly less likely to complete the 18-month questionnaire. Implant loss has been shown to profoundly impact women’s quality of life^47^, so it is possible that the PROs of the PPBR are overestimated here. Furthermore, almost 50% of patients reporting implant loss in this study who did complete the 18-month questionnaire also reported receiving a secondary reconstruction. This may have impacted their scores, but the numbers are too small for any subgroup analysis to be meaningful, and the proportion of women experiencing implant loss in the cohort overall is small. The scores in the Pre-BRA cohort and the recently published clinical reference values for the BREAST-Q in patients having implant-based reconstruction^48^ are similar, thus suggesting they are reflective of the outcomes of implant reconstruction as a whole. Multiple factors are known to impact patient-reported outcomes after breast reconstruction^41,46,49^ and although an exploratory analysis was performed, the cohort was too small to meaningfully undertake a more detailed statistical analysis adjusting for all possible confounders. The aim of this study, however, was exploratory and hypothesis generating and as such it provides valuable insights into the short-term impact of PPBR on PROs. The clinical and PROs of implant-based reconstruction, however, have been shown to change significantly over time^38,46,50^, and therefore studies with longer follow-up will be essential to fully determine the outcomes of the technique, especially in the context of postmastectomy radiotherapy.

There remains an urgent need for high-quality data regarding the short- and long-term impact of PPBR on PROs to support women to make informed decisions about surgery. Ideally comparative data are needed, but the Best-BRA study demonstrated that an RCT comparing pre- and subpectoral techniques was not feasible in the UK due to rapid changes in practice and loss of surgeon equipoise^51^. The OPBC-02/PREPEC trial, an international pragmatic RCT with 372 participants, has successfully accrued and is now in follow-up^52^. The primary outcome is patient-reported chest function assessed with the BREAST-Q at 24 months and therefore the trial will provide important comparative data. However, real-world data involving larger numbers of participants are needed to establish best practice and fully explore the long-term clinical and patient-reported outcomes of PPBR. The ongoing EUBREAST iPREPARE study^53^ is an international prospective PPBR registry with a target recruitment of 1236 women. This large-scale study includes embedded electronic PROs and will allow factors impacting outcomes to be explored and tracked over time.

Evolution of surgical techniques is essential to improve outcomes for patients, but this study adds to the growing body of data to suggest that PPBR fails to deliver on the hypothesized benefits of the technique compared with more established subpectoral procedures. Furthermore, almost half of women are dissatisfied with the ripples/wrinkles in their reconstructed breast, adversely impacting their satisfaction overall. Implant loss rates may also be higher than previously reported, raising ongoing concerns regarding safety. Further large-scale studies exploring how the clinical and patient-reported outcomes of PPBR change over time, particularly regarding late (>3 months) implant loss and the impact of factors such as chest wall radiotherapy on these outcomes are needed. For now, the findings of this study and the uncertainties regarding the long-term outcomes of the technique should be transparently shared with women considering breast reconstruction to support fully informed decision-making based on realistic expectations of the likely results.

## Collaborators

### The Pre-BRA Feasibility Study Steering Group

Peter Barry (Royal Marsden NHS Foundation Trust, London, UK); Simon Cawthorn (North Bristol NHS Trust, Bristol, UK); Matthew Gardiner (Frimley Health NHS Foundation Trust, Frimley, UK); Gareth Irwin (City Hospital, Belfast, UK); Cliona Kirwan (University of Manchester, Manchester, UK); Mairead McKenzie (Patient Advocate, Independent Cancer Patients Voice, London, UK); Shireen McKenzie (Leeds University Teaching Hospital, Leeds, UK); Rachel O’Connell (Royal Marsden NHS Foundation Trust, London, UK); Georgette Oni, Tim Rattay (University of Leicester, Leicester, UK); Pankaj Roy (University Hospitals Oxford NHS Trust, Oxford, UK); Joanna Skillman (University Hospitals Coventry and Warwickshire, Coventry, UK); Soni Soumian (University Hospitals of the North Midlands, Stoke on Trent, UK); Raghavan Vidya (Royal Wolverhampton NHS Trust, Wolverhampton, UK); Lisa Whisker (Worcester Acute Hopsitals NHS Trust, Worcester, UK); and Samantha Williams (Great Western Hospitals NHS Hospitals Foundation Trust, Swindon, UK).

## Supplementary Material

znaf032_Supplementary_Data

## Data Availability

Data are available upon reasonable request. De-identified participant data will be available from the senior author after completion of the study and planned analyses following review by the study steering group. Reuse will be permitted with consent of the study steering group. No additional information will be available.
