# Supplementary material for: Patient-reported outcomes 3 and 18 months after mastectomy and immediate prepectoral implant-based breast reconstruction in the UK Pre-BRA prospective multicentre cohort study
Source: Br J Surg. 2025 Feb 25;112(2):znaf032. doi: 10.1093/bjs/znaf032 (PMC11851068; doi:10.1093/bjs/znaf032)
Supplement: znaf032_Supplementary_Data [file znaf032_supplementary_data.docx]

**Title: Patient-reported outcomes 3- and 18-months after mastectomy and immediate prepectoral implant-based breast reconstruction in the UK Pre-BRA prospective multicentre cohort study**

Authors: Kate L Harvey^1^, Leigh Johnson^2^, Parisa Sinai^1^, Nicola Mills^2^, Paul White^3^, Christopher Holcombe^4^, Shelley Potter^1,5^ on behalf of The Pre-BRA Feasibility Study Steering Group

^1^National Institute for Health Research Bristol Biomedical Research Centre, University Hospitals Bristol NHS Foundation Trust and University of Bristol

^2^Population Health Sciences, Bristol Medical School, Bristol, UK

^3^Applied Statistics Group, University of the West of England, Bristol, UK

^4^Breast Unit, Royal Liverpool University Hospital, Prescot Street, Liverpool, L7 8XP, UK

^5^Bristol Breast Care Centre, North Bristol NHS Trust, Southmead Road, Bristol, BS10 5NB, UK

**Corresponding author.** Shelley Potter Translational Health Sciences, Bristol Medical School. Learning and Research Building, Southmead Hospital, Bristol, BS10 5NB

**ORCID ID: ORCID: 0000-0002-6977-312X Twitter** @drshelleypotter

**Supplementary Materials - Index**

| **Supplementary Results** |  |
| --- | --- |
| Table S1: BREAST-Q scores at baseline, 3- and 18-months | *page 2* |

**Supplementary Results**

**Table S1: BREAST-Q scores at baseline, 3- and 18-months**

|  | **Baseline scores**  **(95% confidence intervals)** | **3-month scores**  **(95% confidence intervals)** | **18-month scores**  **(95% confidence intervals)** |
| --- | --- | --- | --- |
| Satisfaction with Breasts | 63.8 (61.5-66.1) | 66.4 (64.2-68.6) | 60.9 (58.4-63.4) |
| Psychosocial Well Being | 69.8 (67.8-71.9) | 70.6 (68.0-73.2) | 69.6 (66.9-72.4) |
| Sexual Well Being | 58.2 (55.4-61.1) | 52.0 (48.4-55.6) | 48.1 (44.4-51.9) |
| Physical Well Being | 82.8 (81.0-84.7) | 73.5 (71.4-75.6) | 74.7 (72.2-77.2) |
| Animation deformity |  | 78.4 (76.0-80.9) | 75.4 (72.6-78.2) |

**Supplementary Appendixes**

**Supplementary Figures and Tables**

**References**
